# Prevalence of signs and symptoms of laryngopharyngeal reflux in snorers with suspected obstructive sleep apnea

**DOI:** 10.5935/1808-8694.20130105

**Published:** 2015-10-08

**Authors:** Sandra Doria Xavier, Júlio Patrocínio Moraes, Claudia Alessandra Eckley

**Affiliations:** a MSc, Medicine, School of Medical Sciences of the Santa Casa de São Paulo (Second Assistant, Department of Otorhinolaryngology).; b Sixth-year Medical Student, School of Medical Sciences of the Santa Casa de São Paulo (Medical Student).; c PhD, Medicine, School of Medical Sciences of the Santa Casa de São Paulo; Fellow, Professional Voice, Thomas Jefferson University - Philadelphia (Assistant Professor). Santa Casa de São Paulo.

**Keywords:** body mass index, laryngitis, obstructive sleep apnea

## Abstract

Obstructive sleep apnea (OSA) is believed to be correlated with laryngopharyngeal reflux (LPR).

**Objective:**

To study the prevalence of signs and symptoms of reflux in snorers with suspected OSA.

**Method:**

This cross-sectional study enrolled 74 patients assessed positive for OSA with the Berlin questionnaire. The subjects were followed up at the sleep disorder ward of a university center. Studied variables included the BMI, the Epworth sleepiness scale, the reflux symptom index (RSI), the reflux finding score (RFS), and their subdomains related to increased inflammation. The correlations between sleep questionnaires, reflux scales, and their subdomains were deemed statistically significant when *p* < 0.05.

**Results:**

Ninety-eight percent of the subjects had symptoms and signs suggestive of LPR; prevalence was significantly higher among obese individuals (*p* = 0.002).

**Conclusion:**

The significant difference seen in the prevalence of signs of inflammation suggestive of LPR when obese and non-obese subjects with suspected OSA were compared indicates that obesity may affect inflammatory findings of the pharynx and larynx. The high prevalence of symptoms and signs of reflux in patients with suspected OSA calls for more studies on the matter.

## INTRODUCTION

Primary snoring and obstructive sleep apnea (OSA) are highly prevalent in the general population^1^, ^2^, ^3^, ^4^, as is gastroesophageal reflux disease (GERD) and its gastroesophageal manifestation, laryngopharyngeal reflux (LPR)^5^, ^6^, ^7^, ^8^, affecting 20%^1^ and 20%-40%^9^, ^10^ of the adult population respectively. Respiratory sleep disorders have significant impact upon the health and quality of life of patients, and have been correlated with higher risk of automotive accidents caused by diurnal sleepiness or attention deficit^2^, and cardiovascular disease^3^, ^4^. The annual cost of treating the medical consequences of sleep apnea in the United States has been estimated at US$ 3.4 billion^1^. There is no data on the socioeconomic impact of this condition in Brazil. Similarly, laryngopharyngeal reflux may introduce significant morbidity and greatly impact the quality of life of individuals^9^, ^10^.

An association between the two conditions has been suggested in the literature^11^, ^12^, yet it is still unclear whether they share the same risk factors and coexist in the same populations (i.e., overweight adult subjects aged 40 and older)^13^. If such association exists, it is not clear whether reflux episodes are the cause or one of the consequences of apnea or obesity.

Descriptions of the association between laryngopharyngeal reflux and OSA are based on the possible inflammatory impact of gastroduodenal contents upon the pharynx and larynx, causing inflammation and reduction of the lumen of the upper airways^12^, ^14^, in addition to possible local alterations in the mechanoreceptors of the pharynx^12^, ^14^, ^15^ and vagal hyperactivity leading to laryngospasm and coughing^16^.

Obesity is possibly the most common risk factor correlated with these diseases, and may easily help explain apnea and reflux episodes caused by reductions in upper airway patency associated with increased intra-abdominal pressure and reduced intrathoracic pressure^11^, ^12^, ^17^. However, a strictly causal association has not been established yet or even individualized for each of the comorbidities.

Despite the descriptions of a possible correlation between OSA and LPR, population studies designed with this purpose are needed to shed light on the matter. Therefore, this descriptive cohort study aimed to assess the prevalence of symptoms and signs of LPR in snorers with OSA.

## METHOD

This study was approved by the institution's Human Research Ethics Committee and given permit (# 015/12) A total of 170 consecutive patients seen at the sleep disorder clinic of a tertiary care hospital were recruited between May and December of 2012. The following enrollment criteria were applied: age between 21 and 65 years and positive scores in the Berlin Questionnaire^1^, ^3^. The exclusion criteria were as follows: history of smoking or alcohol consumption, history of chronic inflammation or head and neck tumors, and prior digestive tract and/or head and neck surgery. The size of the sample was calculated based on the prevalence of each of the studied diseases in the population in general and the number of patients seen in the sleep disorder clinic of a reference hospital in the city of São Paulo. Calculations indicated that samples of 60 or more subjects would meet the statistical significance requirements for a descriptive cohort study.

All patients underwent physical examination. Subjects had their body mass indexes (BMI) calculated and answered specific questionnaires to assess how likely they were of having OSA and LPR. Laryngoscopy was performed to assess signs of inflammation in the laryngopharyngeal segment. The patients who failed to complete the tests were excluded from the study.

In order to assess the impact of obesity in both conditions, patients were subdivided based on their BMIs into two groups: Group I (non-obese) - subjects with BMI < 30 and Group II (obese) - subjects with BMI ≥ 30 < 40. Morbidly obese patients were not included in the study (BMI > 40).

Cases of suspected obstructive sleep apnea were characterized with the aid of the Epworth sleepiness scale, a validated tool used to assess the chances of patients falling asleep in eight everyday life situations, in which scores greater than 10 have been strongly correlated with OSA^18^.

Similarly, cases of suspected laryngopharyngeal reflux were identified through the analysis of symptoms and signs of disease by two validated instruments, the reflux symptom index (RSI)^19^ and the reflux finding score (RFS)^20^, ^21^. Subjects with RSI scores ≥ 13 and RFS scores ≥ 7 were considered positive for LPR^19^, ^20^, ^21^.

More specific analysis was carried out to study the specific subscales of the scales mentioned above possibly associated with inflammation in the laryngopharyngeal segment and airway patency involvement. The specific variables considered in the RSI were: pharyngeal globus, hawking, and difficulty swallowing. The variables selected in the RFS were: ventricular obliteration, vocal fold edema, diffuse laryngeal edema, and posterior commissure hypertrophy.

For the purposes of statistical calculations, all subscales of the studied symptoms were subdivided into score-based categories: scores of 0, 1, and 2 were ranked as mild/moderate; and scores of 3, 4, and 5 were considered moderate/severe. Laryngoscopy findings such as vocal fold edema, diffuse laryngeal edema, and posterior commissure hypertrophy with scores ranging from 0 to 4 to reflect intensity of involvement were grouped as follows: scores of 0, 1, and 2 were considered mild/moderate; scores of 3 and 4 were considered moderate/severe. In ventricular obliteration, complete obliteration was separated from findings of no obliteration and partial obliteration.

The data sets were treated using *Student's t*-test and the chi-square test for parametric variables. The scores of groups I and II in the Epworth sleepiness scale, RSI, RFS, and their subscales were compared using non-parametric tests (ANOVA, Fischer's exact test, or Mann-Whitney U test). Statistical significance was set for *p* < 0.05.

## RESULTS

Only 74 of the 170 patients recruited initially met the enrollment criteria. Thirty-six patients were females and 38 were males. Female patients had a mean age of 47.7 years (20-64 years) while males had a mean age of 53.8 years (21-63 years). Mean BMI was 31.3 (19.8-42.5) for females and 31.3 (25-41) for males. Given the lack of statistically significant differences between subject ages, BMI, and qualitative variables of male and female patients, the two genders were considered together.

Group I (non-obese) had 36 patients (19 females and 17 males) and Group II (obese) had 38 patients (17 females and 21 males). Group I had a mean BMI of 26.4 (19.8-29.7), while Group II subjects had a mean BMI of 34.4 (30-42.5). The mean score in the Epworth sleepiness scale for Group I subjects was 14.4 (1-24), and 13.1 (3-24) for individuals in Group II. No statistically significant differences were found between the groups of obese and non-obese snorers for diurnal sleepiness as assessed by the Epworth sleepiness scale.

The mean RSI score for Group I subjects was 17 (1-40). Sixty-four percent of the 23 (13 females and 10 males) subjects in this group had scores greater than 13. Group II subjects had a mean RSI score of 21 (0-44), and 79% of the 30 (14 females and 16 males) individuals in this group had scores greater than 13. No statistically significant differences were seen between the RSI scores of both groups. The independent variable in the RSI with the greatest impact upon the final scores of obese and non-obese subjects was hawking, which was not statistically significant.

The prevalence of signs and symptoms suggestive of LPR (RSI and RFS-positive) in the studied population was 89% (66/78), with significantly higher rates seen in group II (68%) when compared to group I (58%) (*p* < 0.05).

**Graph 1:**
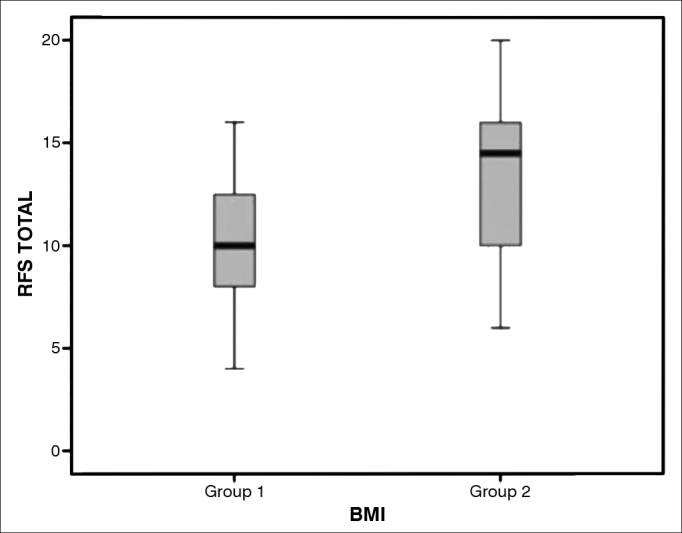
Box plot chart comparing the results from the Reflux Finding Score - RFS among snorers suspected of having obstructive sleep apnea (OSA) non-obese (BMI < 30 - non-obese) and obese (BMI > 30 - Group II).

**Graph 2:**
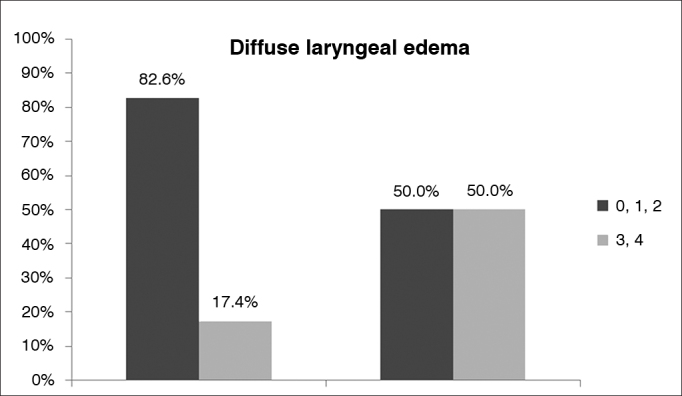
Comparison of results for ‘diffuse laryngeal edema' in the Reflux Finding Score (RFS) of non-obese (BMI < 30 - Group I) and obese (BMI > 30 - Group II) snorers with suspected obstructive sleep apnea (OSA). The dark grey bar represents mild/moderate edema and the light grey bar represents moderate/severe diffuse laryngeal edema.

**Graph 3:**
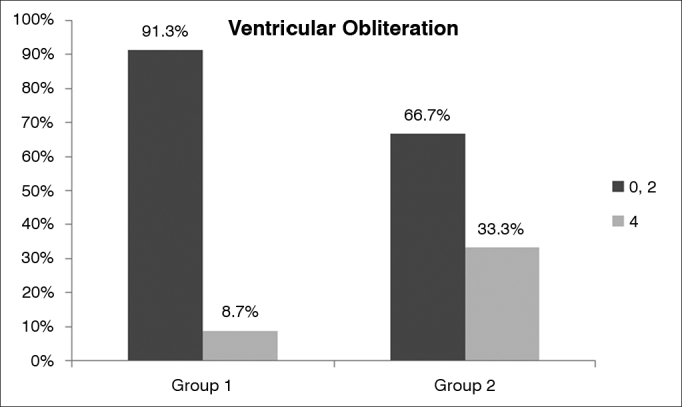
Comparison of results for ‘ventricular obliteration' in the Reflux Finding Score (RFS) of non-obese (BMI < 30 - Group I) and obese (BMI > 30 - Group II) snorers with suspected obstructive sleep apnea (OSA). The dark grey bar represents mild/moderate ventricular obliteration and the light grey bar represents moderate/severe ventricular obliteration.

The RFS scores of individuals in groups I and II were statistically different (*p* < 0.05) (Graph 1). The mean RFS score of group I subjects was 10.1 (4-16), against 13.4 (6-20) of group II patients. In group I, 33 (92%) patients (17 females and 16 males) had RFS scores > 7; in group II, 32 (84%) patients (13 females and 19 males) had RFS scores > 7. When the independent variables of the RFS were considered, both moderate or severe diffuse laryngeal edema and ventricular obliteration were positively correlated with the BMI (*p* = 0.002) (Graph 2 and 3).

**Graph 4:**
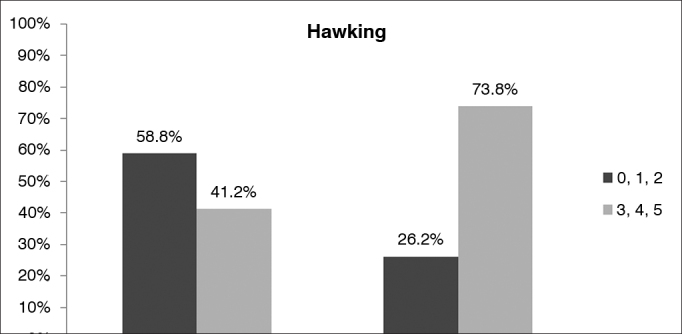
Comparison of results for ‘hawking' in the Reflux Symptom Index (RSI) of sleepy and non-sleepy snorers with suspected obstructive sleep apnea (OSA). The dark grey bar represents mild/moderate hawking and the light grey bar represents moderate/severe hawking.

When patients were compared based on their scores in the Epworth sleepiness scale, the only independent variable of the RSI to show a positive correlation with excessive diurnal sleepiness was hawking (*p* < 0.05). Severe hawking was reported by 73% of the sleepy patients and 41% of the non-sleepy patients (Graph 4).

None of the independent variables of the RFS were statistically correlated with the Epworth sleepiness scale.

## DISCUSSION

Controversy still looms over the clinical diagnosis of laryngopharyngeal reflux, particularly as the signs and symptoms attributed to LPR are also seen in a number of ear, nose, and throat diseases. Therefore, in an attempt to establish an unbiased analysis, it is essential to exclude other known causes of laryngopharyngeal injury. This study had strict exclusion criteria, such as smoking and chronic alcohol intake, rhinosinusitis, and use of medication known to irritate the laryngopharyngeal mucosa.

Obesity is known to be a significant risk factor in OSA and LPR. In addition to the larger abdominal volume and the increased pressure of the abdomen upon the thoracic cavity, reduced upper airway lumen may create inflammatory conditions secondary to the collapse and friction of soft tissue in the upper airways to mimic inflammation findings suggestive of laryngopharyngeal reflux.

Although the prevalence of signs and symptoms suggestive of LPR in this study were very high among obese and non-obese snorers, prevalence rates were significantly higher for patients with a BMI above 30.

Statistically significant differences were also seen in obese patient RFS scores and in diffuse laryngeal edema and ventricular obliteration. It has been suggested that obesity plays a role in the signs of laryngeal inflammation, obese patients have more reflux, or that probably both are true. No positive correlation was established between diurnal sleepiness (Epworth sleepiness scale scores > 10) and RFS scores when obese and non-obese patients were considered.

Hawking was the only independent variable for which the RSI and Epworth scale scores were positively correlated. Although this complaint is very commonly seen in other upper airway inflammatory processes, it was significantly more intense in obese patients and RSI/RFS-positive patients^18^, ^20^. This finding may be associated with reduced upper airway patency and increased tissue laxity, both leading to more significant mechanical trauma.

In 2005, Halum et al.^22^ published a retrospective series comparing the results of prolonged esophageal pH measurements and the BMI of 285 patients with suspected LPR based on the RSI and RFS scores. The authors found significantly higher BMI in patients with GERD and LPR when compared to patients with LPR alone.

We understand the clinical limitations of not objectively assessing apnea and reflux episodes, but these limitations were mitigated as only Berlin Questionnaire-positive patients were included in the study. The Berlin Questionnaire is a proven sensitive instrument used to detect patients at a high risk of having OSA^1^, ^18^. Likewise, the diagnosis of LPR was based on two internationally validated instruments; the sensitivity of RSI scores > 13 and RFS scores > 7 is greater than 94% in the diagnosis of LPR^19^, ^20^.

Although this was an observational cohort study, signs and symptoms of laryngopharyngeal reflux were seen in 89% of the studied population.

More detailed studies are needed to analyze the possible associations between OSA and LPR. These studies should include objective assessment to establish the diagnosis of both conditions to shed light on the factors impacting LPR and OSA. A prospective study with pH measurements and impedance testing combined with polysomnography in patients with this profile is currently in progress.

## CONCLUSION

Eighty-nine percent of the adult patients with obstructive sleep apnea included in this study had signs and symptoms suggestive of laryngopharyngeal reflux. Prevalence of LPR signs and symptoms was significantly higher among obese subjects.

## References

[1] Sleep-related breathing disorders in adults: recommendations for syndrome definition and measurement techniques in clinical research (1999). The Report of an American Academy of Sleep Medicine Task Force. Sleep.

[2] Tregear S, Reston J, Schoelles K, Phillips B (2009). Obstructive sleep apnea and risk of motor vehicle crash: systematic review and meta-analysis. J Clin Sleep Med.

[3] Marin JM, Carrizo SJ, Vicente E, Agusti AG (2005). Long-term cardiovascular outcomes in men with obstructive sleep apnoea-hypopnoea with or without treatment with continuous positive airway pressure: an observational study. Lancet.

[4] Drager LF, Bortolotto LA, Figueiredo AC, Krieger EM, Lorenzi GF (2007). Effects of continuous positive airway pressure on early signs of atherosclerosis in obstructive sleep apnea. Am J Respir Crit Care Med.

[5] Kapur V, Blough DK, Sandblom RE, Hert R, de Maine JB, Sullivan SD (1999). The medical cost of undiagnosed sleep apnea. Sleep.

[6] Koufman JA (1991). The otolaryngologic manifestations of gastroesophageal reflux disease (GERD): a clinical investigation of 225 patients using ambulatory 24-hour pH monitoring and an experimental investigation of the role of acid and pepsin in the development of laryngeal injury. Laryngoscope.

[7] Richter JE (1997). Extraesophageal presentations of gastroesophageal reflux disease. Semin Gastrointest Dis.

[8] Altman KW, Stephens RM, Lyttle CS, Weiss KB (2005). Changing impact of gastroesophageal reflux in medical and otolaryngology practice. Laryngoscope.

[9] Jaspersen D, Kulig M, Labenz J, Leodolter A, Lind T, Meyer-Sabellek W (2003). Prevalence of extra-oesophageal manifestations in gastro-oesophageal reflux disease: an analysis based on the ProGERD Study. Aliment Pharmacol Ther.

[10] Dore MP, Pedroni A, Pes GM, Maragkoudakis E, Tadeu V, Pirina P (2007). Effect of antisecretory therapy on atypical symptoms in gastroesophageal reflux disease. Dig Dis Sci.

[11] Demeter P, Pap A (2004). The relationship between gastroesophageal reflux disease and obstructive sleep apnea. J Gastroenterol.

[12] Eskiizmir G, Kezirian E (2009). Is there a vicious cycle between obstructive sleep apnea and laryngopharyngeal reflux disease?. Med Hypotheses.

[13] Karkos PD, Leong SC, Benton J, Sastry A, Assimakopoulos DA, Issing WJ (2009). Reflux and sleeping disorders: a systematic review. J Laryngol Otol.

[14] Payne RJ, Kost KM, Frenkiel S, Zeitouni AG, Sejean G, Sweet RC (2006). Laryngeal inflammation assessed using the reflux finding score in obstructive sleep apnea. Otolaryngol Head Neck Surg.

[15] Nguyen AT, Jobin V, Payne R, Beauregard J, Naor N, Kimoff RJ (2005). Laryngeal and velopharyngeal sensory impairment in obstructive sleep apnea. Sleep.

[16] Koufman JA (2002). Laryngopharyngeal reflux 2002: a new paradigm of airway disease. Ear Nose Throat J.

[17] Kerr P, Shoenut JP, Millar T, Buckle P, Kryger MH (1992). Nasal CPAP reduces gastroesophageal reflux in obstructive sleep apnea syndrome. Chest.

[18] Johns MW (1992). Reliability and factor analysis of the Epworth Sleepiness Scale. Sleep.

[19] Belafsky PC, Postma GN, Koufman JA (2002). Validity and reliability of the reflux symptom index (RSI). J Voice.

[20] Belafsky PC, Postma GN, Koufman JA (2001). The validity and reliability of the reflux finding score (RFS). Laryngoscope.

[21] Almeida AGP, Saliture TBS, Silva AS, Eckley CA (2013). Translation and cultural adaptation of the Reflux Finding Score into brazilian portuguese. Braz J Otorhinolaryngol.

[22] Halum SL, Postma GN, Johnston C, Belafsky PC, Koufman JA (2005). Patients with isolated laryngopharyngeal reflux are not obese. Laryngoscope.

